# Rare Entrapment of the Deep Peroneal Nerve by a Ganglion Cyst at the Ankle

**DOI:** 10.7759/cureus.87993

**Published:** 2025-07-15

**Authors:** Jochen Gerstner Saucedo, Natalia Coriat, Yudel Tamayo, Jorge Moreno, Carlos E Ramirez, Juan Gerstner, Fabiano Nassar de Castro Cardoso

**Affiliations:** 1 Diagnostic Radiology, University of Colorado Anschutz Medical Campus, Aurora, USA; 2 General Surgery, Clinica Imbanaco, Cali, COL; 3 Diagnostic Radiology, University of Miami Miller School of Medicine, Jackson Memorial Hospital, Miami, USA; 4 Pathology, Clinica Imbanaco, Cali, COL; 5 Orthopedics and Traumatology, Clinica Imbanaco, Cali, COL

**Keywords:** ankle mass, deep peroneal nerve, ganglion cyst, mri, nerve entrapment

## Abstract

Entrapment of the deep peroneal nerve (DPN) is an uncommon yet clinically significant cause of lateral ankle and dorsal foot pain. Among its rare etiologies, ganglion cysts are often overlooked during initial assessments. We present a case of a 56-year-old woman who reported a six-month history of progressive, electric shock-like pain on the dorsum of her left foot, accompanied by a firm mass on the anterolateral ankle. The pain became constant after three months. A physical exam showed a slightly mobile mass and a positive Tinel sign around the DPN. An MRI revealed a cystic lesion compressing the nerve, which led to prompt surgical excision. During the surgery, a hemorrhagic ganglion cyst compressing the DPN was discovered and excised, and nerve decompression was performed. While uncommon, ganglion cysts should be considered in the differential diagnosis for patients exhibiting focal nerve symptoms and soft tissue masses. Timely surgical intervention might help prevent chronic neuropathy and promote functional restoration.

## Introduction

Entrapment of the deep peroneal nerve (DPN) is a rare but potentially reversible etiology of the lateral ankle and dorsal foot pain. The diagnosis is often delayed or overlooked due to vague clinical signs and low prevalence [1]. Although trauma is a frequently reported cause, compression from space-occupying lesions like ganglion cysts remains a rare but significant consideration in the differential diagnosis [2,3]. These cysts, whether intraneural or juxta-articular, can lead to considerable neuropathic pain and functional impairment [2,4].

Early identification and diagnosis are essential for preventing chronic nerve injury and enhancing functional outcomes. Magnetic resonance imaging (MRI) and physical exam maneuvers, including the Tinel sign, are valuable diagnostic instruments to detect peripheral nerve entrapment [5]. This case highlights the importance of considering ganglion cysts in the differential diagnosis and the outcomes following surgical decompression regarding symptom resolution and nerve preservation [4,6].

## Case presentation

A 56-year-old female presented with a six-month history of progressive, electric shock-like pain localized on the dorsum of her left foot. Three months after symptom onset, she noted a palpable mass over the anterolateral portion of her ankle. The pain became constant, interfering with daily activities and prompting a medical evaluation. She denied any history of trauma, systemic illness, or previous orthopedic conditions.

Physical examination revealed a firm, trilobulated, minimally mobile mass measuring approximately 1.5 × 1.0 cm on the anterolateral aspect of the left ankle. Percussion over the area elicited a positive Tinel sign along the course of the DPN. There were no signs of distal sensory or motor deficits, and the neurovascular examination of the foot was unremarkable.

**Figure 1:**
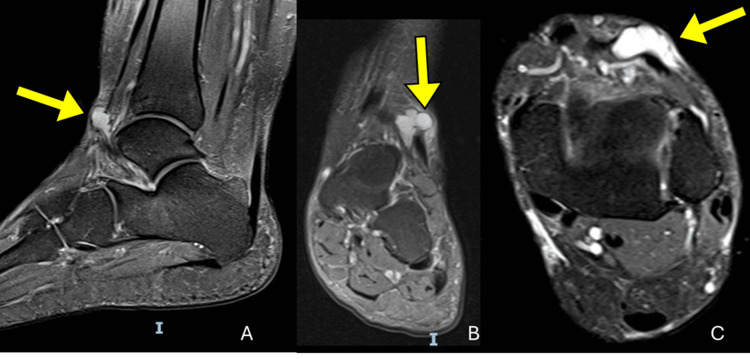
Proton density fat-saturated (PD-FS) MRI of the left ankle demonstrating a ganglion cyst (yellow arrows). (A) Sagittal, (B) coronal, and (C) axial PD-FS images show a well-defined, multilobulated cystic lesion in the anterolateral ankle adjacent to the deep peroneal nerve, superficial to the extensor digitorum longus tendon, also insinuating between the extensor hallucis longus tendon. The lesion exhibits high signal intensity and smooth margins, with no osseous erosion or tendon involvement.

**Figure 2:**
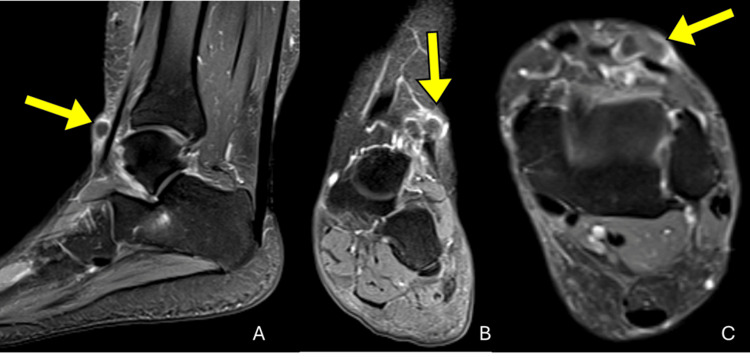
Post-contrast T1-weighted MRI (fat-saturated) of the left ankle showing a pericapsular ganglion cyst (yellow arrows). (A) Sagittal, (B) coronal, and (C) axial planes demonstrate a well-defined, multilobulated lesion with peripheral enhancement, consistent with a ganglion cyst. The lesion is located in the subcutaneous tissues overlying the anterolateral ankle, adjacent to the deep peroneal nerve.

MRI of the left ankle was performed on a high-field scanner, including T1, T2, and short tau inversion recovery (STIR) sequences in orthogonal planes, with and without intravenous gadolinium contrast administration. Imaging revealed a well-circumscribed, multilobulated, cystic lesion located within the subcutaneous tissues superficial to the extensor tendons, measuring approximately 2.5 × 2.0 cm. The lesion exhibited homogeneous peripheral enhancement of its capsule, consistent with a ganglion cyst. No bone erosion, osteochondral injury, or joint effusion was observed. The ligaments, retinacula, and peroneal tendons appeared intact (Figures 1, 2).

The patient underwent surgical excision via an anterolateral approach to the ankle. Intraoperatively, a fluid-filled trilobulated mass containing hemorrhagic synovial fluid was identified compressing the DPN. The mass was excised in its entirety, and the origin traced to the joint capsule. The nerve was decompressed and visualized to be intact; afterwards, the joint capsule was repaired. The patient reported immediate postoperative pain relief. There were no intraoperative or postoperative complications. At early follow-up, she regained normal gait without recurrence of paresthesia. Long-term follow-up is ongoing to monitor for recurrence.

**Figure 3:**
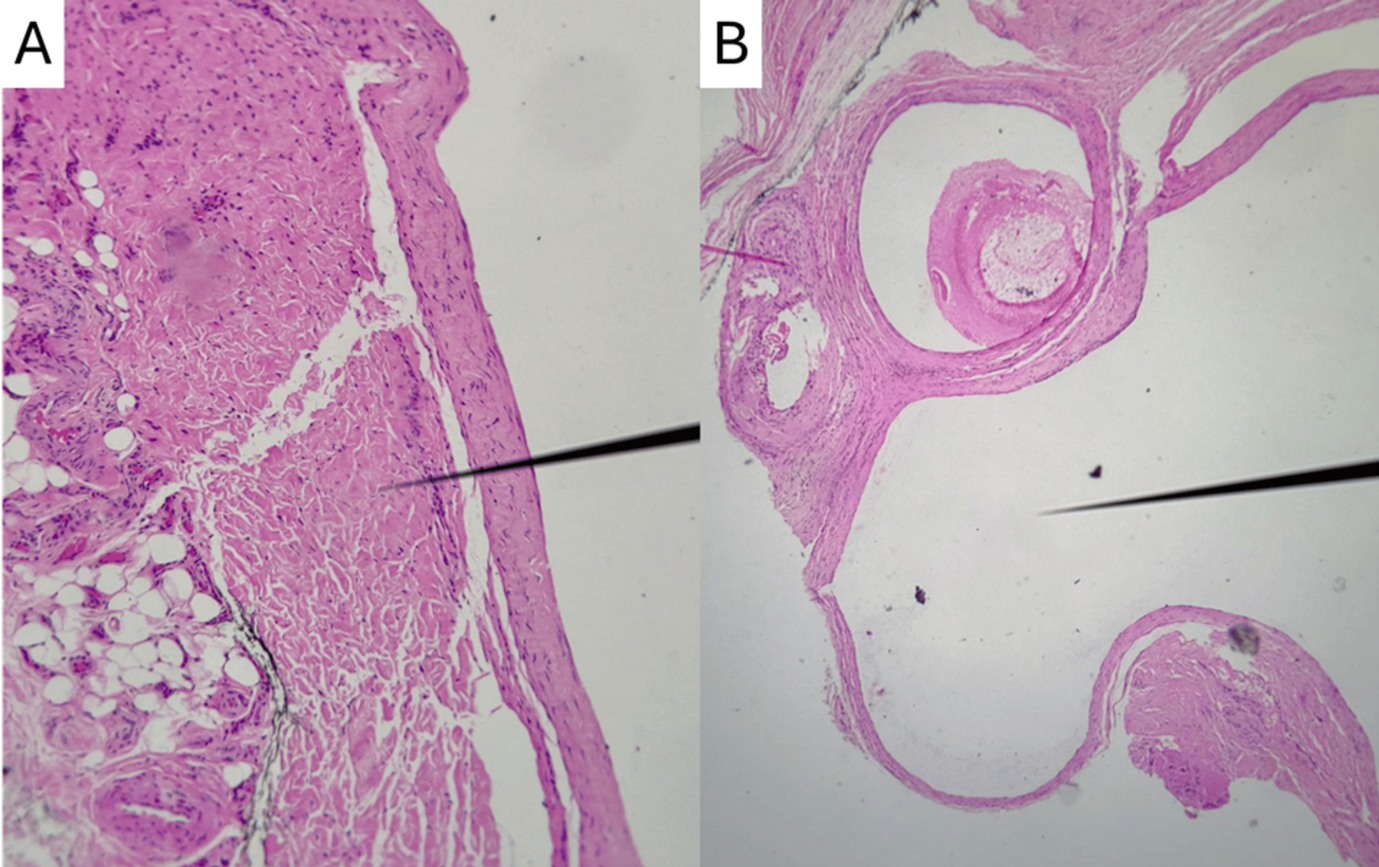
Histologic features of the excised ganglion cyst. Hematoxylin and eosin (H&E)-stained sections show cystic structures within the fibroconnective tissue, lacking an epithelial lining. (A) Dense collagenous stroma surrounding a cleft-like cystic space filled with myxoid material. (B) Low-power view illustrating multiple multiloculated cystic cavities with well-defined fibrous walls and myxoid content. These findings confirm the diagnosis of a ganglion cyst.

Gross pathological examination revealed three irregular, elastic, tan-colored tissue fragments, the largest measuring 1.8 × 1.6 × 0.7 cm. On sectioning, the specimens exhibited multiloculated cystic spaces filled with clear, viscous fluid, consistent with ganglionic contents. The external surface of the largest fragment was inked, and representative sections were submitted for microscopic analysis. Microscopically, the specimen demonstrated fibroconnective tissue containing cystic dilatations lined by myxoid material, but notably lacked any epithelial lining, which is characteristic of a ganglion cyst (Figure 3). There was no evidence of malignancy, inflammation, or nerve sheath tumor.

## Discussion

Entrapment of the superficial peroneal nerve (SPN) is an uncommon but clinically important cause of lateral ankle and dorsal foot pain. Among its rare etiologies, ganglion cysts are frequently overlooked during the initial evaluation. Most ganglion cysts associated with peroneal nerve compression are commonly linked to a history of traumatic knee injury [7]. The patient had no history of direct knee injury, which makes this presentation atypical.

The patient reported experiencing localized pain that was reminiscent of electric shocks and that radiated along the top of the foot. The pain became more intense with the movement of the mass. Clinical evaluation showed a trilobulated soft-tissue mass on the anterolateral aspect of the ankle, along with a positive Tinel's sign in the area innervated by the SPN, suggesting a compressive neuropathy. Clinical diagnosis can often be challenging, considering that ganglion cysts are not usually readily palpable in this area. Furthermore, symptoms associated with ganglion cysts are diverse and nonspecific, potentially manifesting as pain spreading along the path of the compressed nerve, altered sensation, and paresthesias [8].

An MRI was required to characterize the lesion, which was seen as a well-defined, fluid-filled structure arising from the anterolateral aspect of the ankle joint capsule, in close proximity to the SPN. The MRI also provided essential anatomical information for surgical planning, consistent with previous reports that emphasize MRI as a key tool in diagnosing nerve-adjacent cystic lesions [1,3,5]. MRI is considered the gold standard for evaluation since it allows localizing, sizing the lesion accurately, and analyzing the state of the muscles supplied by the peroneal nerve [8]. The imaging findings, alongside the physical assessment, supported the diagnosis of nerve compression caused by a ganglion cyst.

Ganglion cysts, para-articular synovial cysts, and peripheral nerve sheath tumors are potential differential diagnoses for a mass in the area of the SPN [9,10]. Schwannomas and neurofibromas (both peripheral nerve sheath tumors) typically appear as fusiform, solid masses that may cause nerve enlargement. MRI may reveal classic features such as the “split-fat” or “fascicular” sign in schwannomas and the “target” sign in neurofibromas (central low T2 signal with increasing T2 intensity peripherally), but they usually enhance after contrast and do not contain fluid [9,11,12]. In contrast, ganglion cysts (whether intraneural or juxta-articular) present as multilobulated, T2-hyperintense, non-enhancing cystic lesions and may demonstrate communication with neighboring joints. Para-articular cysts, especially those originating from the proximal tibiofibular joint, can also manifest as compressive lesions near the common or superficial peroneal nerve [10]. The imaging features and surgical findings of the lesion in this case suggested a juxta-articular ganglion cyst, absent of intraneural extension.

Nerve sheath myxoma is another rare benign peripheral nerve sheath tumor that should be included in the differential diagnosis for soft tissue masses. Nerve sheath myxomas most commonly present as slow-growing, superficial, multinodular masses in the extremities, particularly the hand, knee, and ankle/foot, and are often painless [13]. On MRI, nerve sheath myxomas appear as well-circumscribed, T2-hyperintense lesions with a myxoid matrix, which can resemble cystic structures due to their high water content. However, unlike ganglion cysts, they often demonstrate variable contrast enhancement and a multinodular architecture, sometimes with an identifiable entering or exiting nerve [14]. Definitive diagnosis requires histopathological evaluation, which reveals S-100 and GFAP positivity, confirming Schwann cell origin. Importantly, these tumors have a high local recurrence rate if not completely excised [13,15].

The preferred treatment for SPN compression caused by ganglion cysts is the surgical excision of the ganglion with meticulous preoperative planning and precise delineation of the lesion. To date, no authors have previously recommended conservative treatment, as surgical treatment has been shown to be successful in most cases when it is performed in its early stages [7,16]. If surgery is delayed, intraneural growth and invasion may cause irreversible axonal damage, leading to significant neuropathic pain and functional impairment [7].

In this case, intraoperative examination confirmed the presence of a ganglion cyst containing hemorrhagic joint fluid, causing displacement and compression of the SPN. Although the nerve remained anatomically intact, it exhibited indentation from the adjacent cyst wall. The lesion was excised entirely, and decompression of the nerve was performed, followed by closure of the joint capsule to decrease the risk of recurrence. A definitive diagnosis was made by histopathological examination, which revealed fibroconnective tissue with cystic cavities that contained myxoid material and lacked an epithelial lining [17,18].

The treatment approach, in this case, focused on alleviating nerve compression while simultaneously addressing the cyst’s articular origin to prevent future enlargement or recurrence. Surgical excision of ganglion cysts, especially when symptomatic and space-occupying, remains the gold standard of care and is associated with excellent functional outcomes in the literature [2,4,6,19]. In this patient, surgical treatment led to rapid postoperative relief, without neurological deficits and early return to normal function.

## Conclusions

This case demonstrates an unusual presentation of a slowly expanding ganglion cyst compressing the DPN, highlighting the significance of integrating clinical examination, imaging studies, and surgical findings to achieve an accurate diagnosis along with appropriate medical treatment. Deep peroneal nerve entrapment caused by a ganglion cyst is a rare but treatable condition. Clinicians should be cautious when patients approach with unexplained dorsal foot discomfort and an accompanying soft-tissue mass. MRI is essential to establish an early diagnosis and to enhance the chance of a complete recovery.
